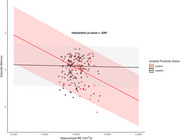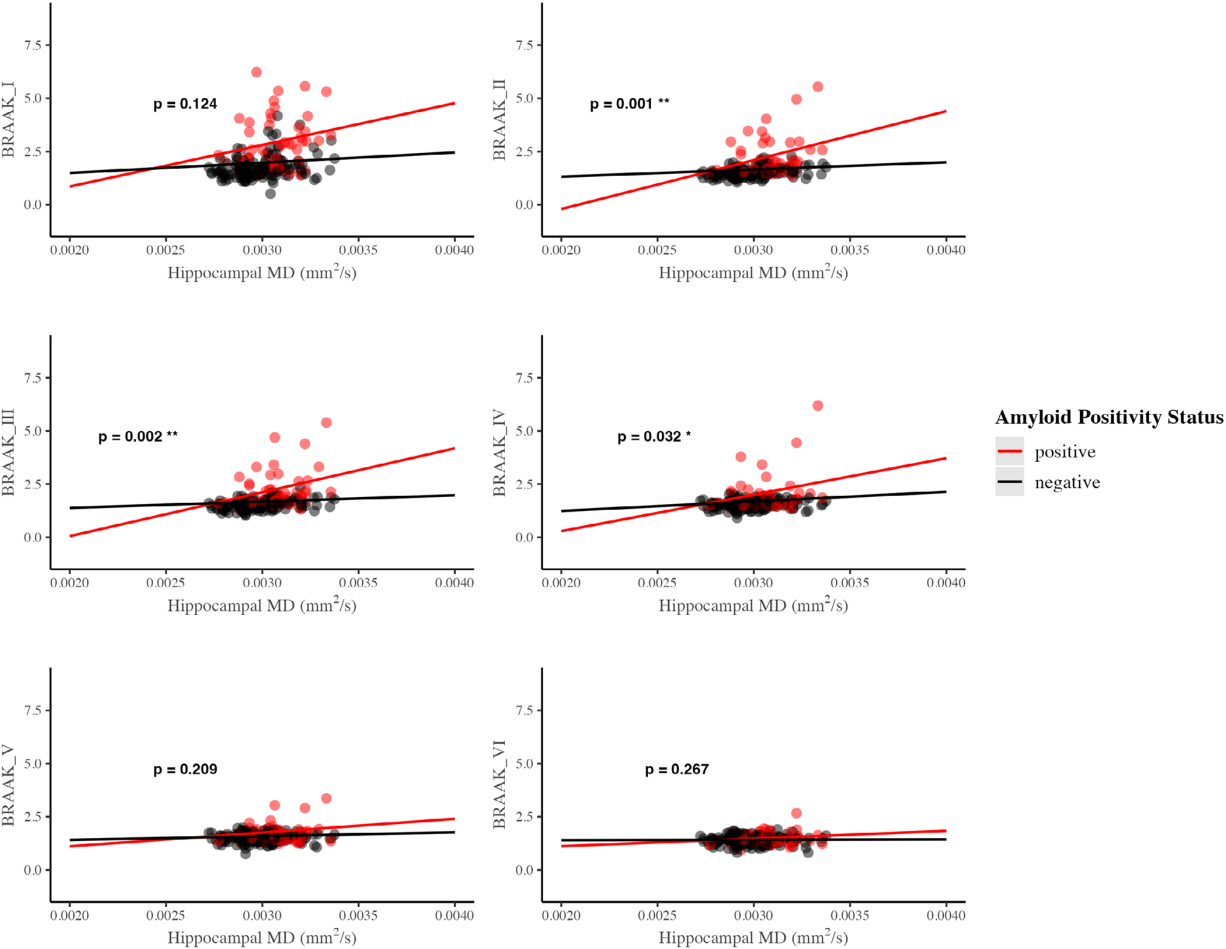# Tau PET Burden is Associated with Hippocampal Microstructure and Episodic Memory in Older Adults with Amyloid Burden

**DOI:** 10.1002/alz70856_097805

**Published:** 2025-12-24

**Authors:** Daniel D. Callow, Nisha Rani, Kylie H. Alm, Corinne Pettigrew, Anja Soldan, Michael I Miller, Marilyn S. S. Albert, Arnold Bakker, Sara Sheikhbahaei, BIOCARD Research Team

**Affiliations:** ^1^ Johns Hopkins University School of Medicine, Baltimore, MD, USA; ^2^ Department of Neurology, Johns Hopkins University School of Medicine, Baltimore, MD, USA; ^3^ Johns Hopkins University, Baltimore, MD, USA; ^4^ Johns Hopkins University Whiting School of Engineering, Baltimore, MD, USA; ^5^ John's Hopkins University School of Medicine, Baltimore, MD, USA; ^6^ Department of Psychiatry and Behavioral Science, Johns Hopkins University School of Medicine, Baltimore, MD, USA; ^7^ Johns Hopkins School of Medicine, 21287, MD, USA

## Abstract

**Background:**

Growing evidence suggests that hippocampal gray matter microstructure, assessed through diffusion‐weighted imaging (DWI), is a sensitive marker of neurodegeneration in Alzheimer's disease (AD). While hippocampal atrophy is a characteristic feature of AD, microstructural changes may precede macrostructural changes such as volumetric loss, offering important insights into the early phases of disease.

**Method:**

This study assessed the relationships between hippocampal microstructure (assessed with DWI) tau pathology [by positron emission tomography (PET)], and episodic memory performance, focusing on the moderating role of Aβ status assessed by PET imaging. The study included 192 participants without dementia (14 with mild cognitive impairment [MCI]) from the BIOCARD cohort (mean age = 68), of which 52 (27%) were Aβ positive. Multiple linear regression analyses tested whether Aβ status moderated associations between hippocampal mean diffusivity (MD), Braak‐staged tau accumulation on PET, and an episodic memory composite score.

**Result:**

Increased hippocampal MD was associated with worse memory (β = ‐0.24, SE = 0.12, *p* =  0.039) and greater tau PET burden in Braak stages II‐IV (β = .29, SE = .08, *p* < 0.001; β = .26, SE = .08, *p* =  0.002; β = .19, SE = .08, *p* =  0.018 for Braak stages II‐IV respectively), but only in individuals who were amyloid positive (e.g., significant amyloid x hippocampal MD interactions). Building on prior findings linking early Braak‐staged tau to memory, we further assessed whether tau PET burden mediated the relationship between elevated hippocampal MD and poorer memory performance. Tau PET burden in Braak stages II‐IV was found to fully mediate the relationship between elevated hippocampal MD and poorer memory performance (indirect effects with 95% CI: β = 0.25 [‐0.47, ‐0.04]; β = 0.19 [‐0.38, ‐0.01]; β = 0.22 [‐0.43, ‐0.02]), but only in amyloid‐positive participants and independent of hippocampal volume. These associations were not significant when excluding MCI participants from the analysis.

**Conclusion:**

These findings suggest hippocampal microstructure may be sensitive to AD‐related pathological burden and associated neurodegeneration, particularly in the early symptomatic phase, and is associated with tau PET and cognitive decline while accounting for hippocampal volume.